# Characterisation of *Aspergillus fumigatus* Endocytic Trafficking within Airway Epithelial Cells Using High-Resolution Automated Quantitative Confocal Microscopy

**DOI:** 10.3390/jof7060454

**Published:** 2021-06-07

**Authors:** Nagwa Ben-Ghazzi, Sergio Moreno-Velásquez, Constanze Seidel, Darren Thomson, David W. Denning, Nick D. Read, Paul Bowyer, Sara Gago

**Affiliations:** 1 Manchester Fungal Infection Group, Division of Infection, Immunity and Respiratory Medicine, Faculty of Biology, Medicine and Health, School of Biological Sciences, The University of Manchester, 2nd Floor, 46 Grafton Street, Manchester M13 9NT, UK; nagwaben@gmail.com (N.B.-G.); s.moreno.velasquez@gmail.com (S.M.-V.); dr.constanze.seidel@gmail.com (C.S.); darren.thomson@manchester.ac.uk (D.T.); david.denning@manchester.ac.uk (D.W.D.); nick.read@manchester.ac.uk (N.D.R.); 2 Faculty of Medical Technology-Benghazi, National Board for Technical & Vocational Education, Libyan Ministry of Higher Education and Scientific Research, Al-Salmani, Benghazi 17091, Libya; 3 Centre for Organismal Studies (COS), Heidelberg University, Im Neuenheimer Feld 230, 69120 Heidelberg, Germany

**Keywords:** *Aspergillus fumigatus*, airway epithelial cells, phagocytosis

## Abstract

The precise characterization of the mechanisms modulating *Aspergillus fumigatus* survival within airway epithelial cells has been impaired by the lack of live-cell imaging technologies and user-friendly quantification approaches. Here we described the use of an automated image analysis pipeline to estimate the proportion of *A. fumigatus* spores taken up by airway epithelial cells, those contained within phagolysosomes or acidified phagosomes, along with the fungal factors contributing to these processes. Coupling the use of fluorescent *A. fumigatus* strains and fluorescent epithelial probes targeting lysosomes, acidified compartments and cell membrane, we found that both the efficacy of lysosome recruitment to phagosomes and phagosome acidification determines the capacity of airway epithelial cells to contain *A. fumigatus* growth. Overall, the capability of the airway epithelium to prevent *A. fumigatus* survival was higher in bronchial epithelial than alveolar epithelial cells. Certain *A. fumigatus* cell wall mutants influenced phagosome maturation in airway epithelial cells. Taken together, this live-cell 4D imaging approach allows observation and measurement of the very early processes of *A. fumigatus* interaction within live airway epithelial monolayers.

## 1. Introduction

More than 10 million people globally suffer from lung diseases caused by the environmental fungus *Aspergillus fumigatus* [1], and over 200,000 annually die from it [2]. Inhalation of *A. fumigatus* spores is constant and unavoidable for all human beings [3]. Early after exposure, *A. fumigatus* spores are eliminated from the airways by the innate lung defences [4,5,6]. However, the extended use of immunosuppressive treatments alongside the extremely large population suffering from chronic lung conditions and severe respiratory infections has placed *A. fumigatus* as the major respiratory mould pathogen in patients with debilitated lung defences [7,8,9,10,11].

Intraphagosomal killing of *A. fumigatus* spores by professional phagocytes such as macrophages is the most efficient process to prevent fungal persistence and the development of disease [12,13,14,15]. However epithelial cells vastly outnumber macrophages in the normal lung and an increasing body of evidence has shown that uptake of adherent or opsonised spores by the airway epithelium materially contributes to the digestion of *Aspergillus* spores within matured phagolysosomes [16,17,18,19]. The mechanism by which the lung epithelium kills *A. fumigatus* spores is poorly understood. Several studies have indicated this process is critical for the release of immune modulators which attract and recruit professional phagocytes to the site of infection to prevent establishment of infection [20,21,22,23,24,25].

Airway epithelial cells have been shown to efficiently take up fungal spores in a timely manner [26,27,28,29,30,31,32]. In order to prevent fungal spore germination and escape, phagosomes containing internalized spores are acidified and fused with lysosomes [33,34,35]. Although potentially efficient in fungal clearance, previous work by us and others found that during early stages of infection, less than 3% of *A. fumigatus* spores can survive within the phagolysosome and escape by hyphal elongation without causing any significant host damage in a non-lytic egress mechanism [19,35]. However, several respiratory pathogens have developed anti-phagocytic strategies which allow them to survive within the airways and cause disease [36,37]. We therefore surmised that *A. fumigatus* pathogenicity can be driven by impairing epithelial endocytic pathways. These could involve spore recognition, phagosome acidification or phagosome fusion with lysosomes.

There is substantial evidence that *A. fumigatus* can manipulate phagosome acidification by expressing 1,8-dihydroxynaphthalene-melanin [16,38,39]. In fact, *A. fumigatus pksP* deletion mutants lacking this pigment are more susceptible to being killed within acidified phagosomes of professional phagocytes in vitro, and display attenuated virulence in in-vivo models of disease [39,40]. Additionally, to avoid host recognition, *A. fumigatus* surfaces are covered by a monolayer of hydrophobins, which prevent melanin exposure [41]. *Aspergillus* rodletless mutants are more susceptible in recognition and killing by both alveolar macrophages and dendritic cells, although this is not linked to increased phagosome acidification by these host cell effectors [42,43]. However, little is known about the dynamics of the fungal factors involved in early phase survival within the lung epithelium phagosome [44]. Additionally, the efficiency of the epithelial endocytic pathway to stifle fungal growth likely differs by tissue or cell type, as seen by lipopolysaccharide and pollutants, which activate bronchial and alveolar epithelial cells by different mechanisms [45,46].

Quantification of phagosome maturation during *A. fumigatus* infection typically relies on either the use of fixed samples, which halt and can alter the endocytic process, or cellular reporters which cannot be easily transferred between different cellular systems. Here, we explore the suitability of high-resolution confocal microscopy approach to define the dynamics of phagosome acidification and maturation within the lung epithelium in preventing fungal survival. We found differences in spore recognition and phagolysosome maturation across the most commonly used lung epithelial cell lines.

## 2. Materials and Methods

### 2.1. Fungal Strains

*Aspergillus fumigatus* strains (Table S1) were cultured at 37 °C for 48–72 h on Sabouraud dextrose agar (Sigma-Aldrich, Gillingham, UK). Spores were harvested using PBS-Tween 20 at 0.1%, and the concentration of spores was determined using a haemocytometer.

### 2.2. Cell Lines

Human pulmonary carcinoma alveolar epithelial cell line A549 (ATCC CCL-185) and 16HBE bronchial epithelial cells [47] were used throughout this study. 16HBE were a kind gift of Dr D. Gruenert (San Francisco, CA, USA). For all experiments, cells were maintained at 37 °C, 5% CO_2_ in supplemented Dulbecco’s Modified-Eagle’s Medium (DMEM, Sigma-Aldrich, Gillingham, UK) or Minimum Essential Medium Eagle (MEM, Sigma-Aldrich, Gillingham, UK) for A549 and 16HBE cells respectively. Supplemented media for A549 cells consisted of 10% Foetal Bovine Serum (FBS, Sigma-Aldrich, Gillingham, UK) and 1% Penicillin-Streptomycin (Sigma-Aldrich, Gillingham, UK) in DMEM. To grow 16HBE cells, MEM was supplemented with 10% FBS, 1% Penicillin-Streptomycin and 1% L-glutamine (Sigma-Aldrich, Gillingham, UK). For all experiments, 10^5^ epithelial cells were seeded in 24-glass bottom plates (Greiner Bio-One SensoPlate™, Kremsmünster, Austria) and incubated until confluence was 80–90%, unless otherwise described. Live cell imaging was performed using supplemented phenol red-free DMEM/F12 Medium (Thermo Fisher Scientific, Waltham, MA, USA) [35].

### 2.3. Quantification of the Number of Lysosomes during A. fumigatus Infection of Lung Epithelial Cells

In order to determine the stages of phagosome-lysosome fusion, sub-confluent (70%) monolayers of 16HBE and A549 epithelial cells were labelled with CellLight Lysosome-GFP BacMam 2.0 marker (Life Technologies, Carlsbad, CA, USA) according to the manufacturer’s instructions, and further incubated for 16 h at 37 °C, 5% CO_2_. Confluent lysosome-labelled monolayers were then challenged with 10^6^ spores of A1163 (pyrG-::βtub-eGFP,pyrG+; strain: MFIGGFP4) for 3 h. At that time, extracellular spores were killed by incubation with 25 μg/mL of nystatin (Sigma-Aldrich, Gillingham, UK) for 1 h in culture media [27]. Cytoplasmic phagosome and epithelial cell membranes were then stained with Cell Mask Deep Red (Life Technologies, Carlsbad, CA, USA) according to manufacturer’s instructions, and the number of lysosomes per cell was determined using live-cell high-resolution confocal microscopy at 10 min intervals from 5 to 6 h post-infection. Additionally, the number of lysosomes per epithelial cell containing either *A. fumigatus* spore or germlings within phagosomes or phagolysosomes was comparatively analysed at 16 h post-infection.

### 2.4. Quantification of the Rate of Lysosome—Phagosome Fusion during A. fumigatus Infection of Epithelial Cells

To further characterize the importance of phagosome-lysosome fusion in preventing *A. fumigatus* germination, the rate of lysosome fusion to phagosomes was measured by determining the amount of GFP-LAMP1 fluorescence transferred from lysosomes into the developing phagolysosomal membrane in both A549 and 16HBE cells containing *A. fumigatus* spores and germlings from 4 to16 h post-infection. Lysosomes and plasma membranes of epithelial cells were labelled and challenged with *A. fumigatus* MFIGGFP4 spores, as described above. As lysosome fusion defines phagolysosome differentiation from the phagosome, we were also able to utilise datasets generated during this *A. fumigatus* epithelia infection time course to determine differences in the proportion of *A. fumigatus* germlings and spores within phagosomes (CellMask positive, LAMP-1 negative) and phagolysosomes (CellMask positive, LAMP-1 positive), as a means to determine whether the phagosome/phagolysosomal killing mechanism was effective. Additionally, to investigate if phagosome maturation had a negative impact on *A. fumigatus* germination, the time of spore germination of *A. fumigatus* spores contained in phagosomes or phagolysosomes was determined on datasets acquired during this time course.

### 2.5. Determining A. fumigatus Viability within Phagolysosomes by Confocal Microscopy

To further describe the development and fate of internalized spores within A549 and 16HBE lung epithelial cells, the proportion of internalized spores, which were digested, remained resident, swelled or germinated within phagolysosomes was determined at 24 h post-infection. Digested spores were scored by the assessment of loss of fungal cytoplasmic RFP fluorescence; resident spores were scored as those preserving the same size and fluorescence as the original spore inoculum, while swollen spores were those showing increased volume compared to the original inoculum with comparable fluorescence. Germinated spores were counted when a germ tube of >0.5 spore diameters was visible [35].

### 2.6. Assessing the Impact of A. fumigatus Cell Wall Components on Phagosome-Lysosome Fusion Rates in the Lung Epithelium

To determine if *A. fumigatus* cell wall components can modulate the rate of phagosome-lysosome fusion, LAMP-1 labelled 16HBE and A549 monolayers were challenged with *A. fumigatus* A1160; Δ*rodA*, Δ*rodB* and Δ*pksP* mutants. Prior to infection, spores were stained with 200 μM of FUN-1 viability stain (Life Technologies, Carlsbad, CA, USA) in 2% glucose, 10 mM HEPES. The percentage of FUN-1 labelled spores within phagolysosomes (CellMask positive, LAMP-1 positive, shown in green) or phagosomes (CellMask positive, LAMP-1 negative, shown in magenta) was evaluated at 3 h post-infection.

### 2.7. Determining Synchronization of Phagosome Acidification and Phagosome Maturation upon A. fumigatus Infection of the Lung Epithelium

To interrogate whether phagosome acidification is synchronized with phagosome-lysosome fusion and depends upon fungal cell wall integrity, the proportion of A1160, Δ*rodA*, Δ*rodB* and Δ*pksP* within acidified or non-acidified phagosomes was determined at 3 h post-infection. A549 and 16HBE confluent monolayers were challenged with 10^6^ fungal spore/mL of A1160 and cell wall deficient FUN-1 fluorescence labelled *A. fumigatus* strains in serum-free media as described above. After incubation, monolayers were incubated with 50 nM of Lysotracker TM red DND-99 Special (Invitrogen, Darmstadt, Germany) for 30 min at 37 °C, 5% CO_2_ in cell culture media, and the plasma membrane was then stained using Cell Mask Deep Red, as described above.

### 2.8. Fungal Killing Assays

In order to quantify the percentage of killed intracellular spores, A549 and 16HBE monolayers were co-incubated with 10^7^ spores/mL of *A. fumigatus,* A1160 and the three cell wall mutants used in this study, at 37 °C with 5% CO_2_ for 6 h. The infected cells were then washed twice with pre-warmed PBS to remove unbound spores, scraped and sonicated. To identify killed spores, propidium iodide (PI, Sigma-Aldrich, Gillingham, UK) was added to sonicated samples at a final concentration of 5 μg/mL and imaged using confocal microscopy (details below in Section 2.10).

### 2.9. siRNA Treatment

To investigate the role of vATPase in phagosome maturation, sub-confluent A549 lung epithelial cells were transfected with 500 pg of siRNA ATP6V0A2 (Ambion, Austin, TX, USA) using Lipofectamine 3000 (Invitrogen, Darmstadt, Germany) according to the manufacturer’s instructions. Cells at 48 h post-transfection were used for infection experiments and changes in the expression of vATPAse were evaluated by qPCR using SensiFAST Syber (Bioline, Memphis, TN, USA) according to the manufacturer’s instructions. Validated primers targeting ATP6V0A2 and RPL13A were purchased from Bio-rad (Hercules, CA, USA).

### 2.10. Image Acquisition and Data Analyses

Confocal images were acquired using a fully motorised Leica SP8x laser scanning confocal microscope equipped with a 63× (1.2 NA) HC PLAN APO UVIS CS2 water immersion objective and a 63× (NA 1.4) HC PLAN APO CS2 oil immersion objective. A pinhole of one airy unit was used for 3D sectioning. Imaging was performed at 37 °C for live-cell imaging. Fluorescence imaging was performed using the excitation/emission conditions recommended by the manufacturers: Cell Mask Deep Red stain (Excitation = 649 nm, Emission = 666 nm HyD/PMT detection window); CellLight Lysosomes-GFP BacMan (Excitation = 488, Emission = 500 nm HyD/PMT detection window); LysoTracker Blue (Excitation = 405, Emission = 422 nm HyD/PMT detection window); FUN-1 (Excitation = 488, Emission = 500 nm HyD/PMT detection window). From 15 to 25 3-D stacks were taken in biological and technical triplicates.

Analysis of 3D and 4D confocal images were performed using IMARIS v8.1.1 (Bitplane, Oxford Instruments, Abingdon, UK). Leica confocal images (.lif) were converted into .ims files using the IMARIS file converter ×64 8.2.0 and imported into IMARIS for 4D segmentation. Each of the four fluorescent confocal volumes (*A. fumigatus*, lysosomes, acidic compartments and epithelial membranes) were loaded into IMARIS. For quantitative analyses, the number of lysosomes were calculated from each image volume by rendering the cytoplasmic membrane (using the “new surface option” function) and the lysosomes (spot object option). Analyses were performed on volumes at 10 min intervals for 1 h at from 5 to 6 h post-infection. Cells were then individually assessed for containing single spores or germlings within their phagosomes and/or phagolysosomes using Fiji. Spores were also automatically assigned intracellular or extracellular identities via the IMARIS XT “split into surface objects” function using MatLab for IMARIS [48]. This function automatically analyzed whether each rendered ‘spot’ (spores and lysosomes) was located within the host cell rendered ‘surfaces’ (epithelia membrane) or not. The statistical raw data, including the number of lysosomes per cell, were exported to excel files using the Vantage function.

To further determine whether the internalized spores were located inside epithelia, the number of spores ‘spots’ co-localized inside phagolysosomes, acidic organelles or the cytosol (not contained within a phagosome or phagolysosome) was analyzed using the same “split into surface objects” function as above, but for acidic niche or phagolysosome membrane rendered ‘surfaces’ in IMARIS. Individual spore ‘spots’ were each assigned an identity: localized in phagosomes, phagolysosomes or acidic phagolysosomes. Raw quantitative data and mean GFP-LAMP1 fluorescence intensity around phagocytic membranes was exported using the Vantage function. To measure and determine differences in spore volumes and surface area over time within phagolysosomes, fluorescent spores in those environments were “surface” rendered and tracked over time (12 h) in IMARIS. Surface rendering for all spores were performed by entering the derived largest spore diameter (5 µm; measured via the line measurement tool in the IMARIS Slice Viewer) into the local contrast background subtraction option, to identify all fluorescent spores of that diameter and below, eliminating any larger fluorescent germling structures. The increase in volume and surface area of tracked spores were then analyzed in IMARIS Vantage mode, where tracked spore volume over time displaying no change were deemed dormant, and those which increased were deemed swollen [35]. The axial imaging resolution of 0.5–1 μm was sufficient to resolve two overlapping spores. Accuracy of automated quantifications was validated by manual counts using a representative subset of images. For that, spores included within phagolysosomes or acidic compartments by both methods, spores excluded from these compartments by both methods, spores within phagolysosomes or acidic phagosomes considered only by one or the other method were scored.

### 2.11. Statistical Analyses

Statistical analyses were performed using GraphPad Prism, v8.0 (GraphPad Software, La Jolla, CA, USA) considering *p* < 0.05 as a cut-off for statistical significance. The reduction in the number of lysosomes per cell min^−1^ was estimated by fitting linear regression. Differences in germination time across conditions, and in fusion rate, or acidification rates or killing efficiency between fungal mutants were estimated by one-way ANOVA using Dunnet´s test for corrected multiple comparison tests, after data normality testing. Differences in proportions were estimated by a Fisher Exact test. An unpaired *t*-test was used to determine differences in the mean intensity of GFP-LAMP1 fluorescence in the phagolysosomes membrane for each cell line. All experiments were performed in biological and technical triplicates, and a minimum of 1000 spores per condition were counted for all experiments. The percentage of agreement between automated and manual counts to identify spores within phagolysosomes or acidic phagosomes was calculated by using the Cohen’s kappa test on GraphPad.

## 3. Results

### 3.1. Rate of Lysosome Recruitment to the Phagosome Determines A. fumigatus Fate within the Lung Alveolar Epithelial Cells

**Figure 1:**
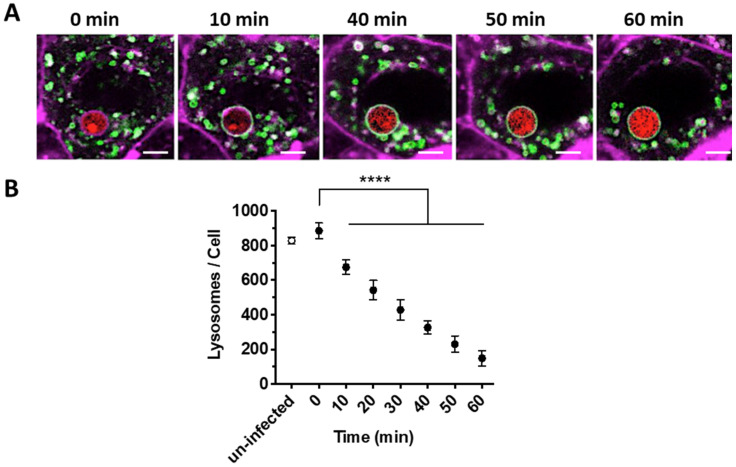
Dynamics of phagosome-lysosome fusion observed during *A. fumigatus* phagocytosis. (**A**) Representative time-lapse confocal images from the central focal plane of a single *A. fumigatus* spore (red) engulfed and localized within A549 alveolar epithelial cell monolayer (magenta) from 5 to 6 h post-infection in 10 min intervals. (**B**) Number of lysosomes within airway epithelial cells which have taken up *A. fumigatus* spores is reduced over time. (mean ± SD of three biological replicates assessed in technical triplicates). Scale Bar = 5 μm. (**** *p* < 0.0001, compared to 0 min). Magenta = epithelial cells membrane (CellMask Deep Red), Green = lysosomes (GFP-LAMP), red = *A. fumigatus* (MFIGRag29, Table S1).

To quantify lysosome trafficking to phagosomes containing *A. fumigatus* spores, the expression of a GFP construct fused to the lysosomal marker LAMP1 was monitored for 1 h in A549 lung epithelial cells (from 5 to 6 h post-infection, Figure 1A). At 5 h post-infection, there were no differences in the number of lysosomes between uninfected cells or those containing *A. fumigatus* spores within phagosomes (829 ± 30 vs. 886.2 ± 59; *p* > 0.05, Figure 1B and Figure S1). During the next 60 min (5–6 h post infection), lysosomes (LAMP1-GFP) progressively translocated from the cytoplasm and fused to the phagosome containing spores (Figure 1A) at a fusion rate of 11.85 ± 0.45 lysosomes/minute.

**Figure 2:**
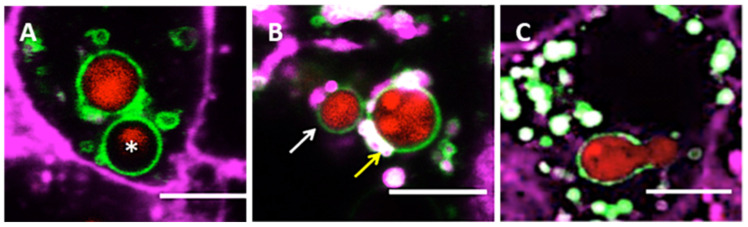
*A. fumigatus* fate within phagolysosomes of A549 alveolar epithelial cells at 18 h post-infection. (**A**) Digested spore (asterisk). (**B**) Resting (white arrow) and swollen spore (yellow arrow) inside the phagolysosome. (**C**) Germinated spore within phagolysosome. Scale Bar = 5 μm. Magenta = airway epithelial cells membrane (CellMask Deep Red), Green = lysosomes (GFP-LAMP), red = *A. fumigatus* (MFIGRag29, Table S1).

Since a small proportion of *A. fumigatus* spores can germinate and escape from the host cell [35], it is likely that survival of spores and germlings within phagolysosomes is determined by both, the number of free lysosomes present in the epithelial cell cytoplasm and their rate of fusion with the phagosome. To monitor the importance of phagosome-lysosome fusion during *A. fumigatus* morphogenesis, the development and fate of internalized spores within phagolysosomes was tracked using live-cell confocal microscopy. Overall, we observed different fates of *A. fumigatus* spores and germlings within phagolysosomes: spores either underwent digestion, remained intact but dormant, became swollen and germinated within the phagolysosome (Figure 2). Significantly, spore germination occurred in those individual epithelial cells which contained a reduced number of lysosomes fusing with the spore-containing phagosome. This indicates that a defined rate of lysosome-phagosome fusion (11.85/min) is required to prevent spore germination (Figure 2C).

**Figure 3:**
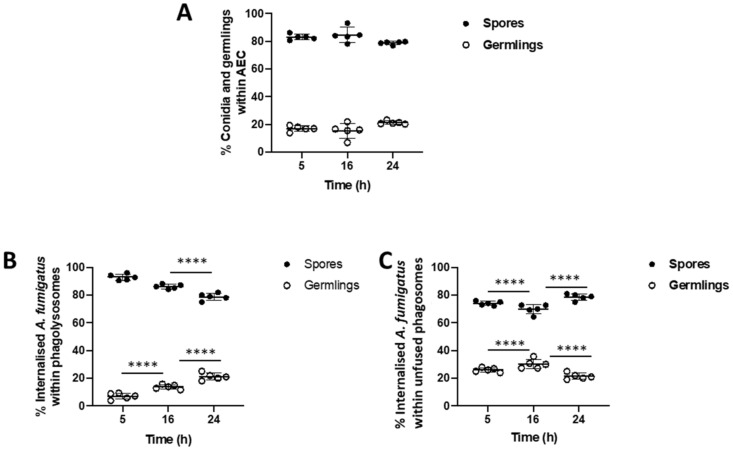
Suboptimal lysosome fusion permits *A. fumigatus* germination within the airway epithelium. (**A**) Proportion *of A. fumigatus* spores and germlings within A549 alveolar epithelial cells. (**B**) Proportion of internalized spores and germlings within phagolysosomes of A549 cells. (**C**) Proportion of internalized spores and germlings within unfused phagosomes of A549 cells. Data represents mean and standard deviation of five biological replicates. Differences in proportions between time points for spores and germlings were independently tested using the Fischer Exact Test. **** *p* < 0.0001.

To further characterize the effect of phagosome-lysosome fusion on the germination of internalized *A. fumigatus* spores, the percentage of internalized spores and germlings within epithelial cells was analyzed over a 24 h challenge live-cell confocal imaging experiment (Figure 3A). The number of spores within phagolysosomes are significantly reduced over time (93.49% at 5 h; 85.69% at 16 h; 78.56% at 24 h), as a concurrent increase in the percentage of germination occurs (6.37% at 5 h to 21.44% at 24 h; *p* < 0.0001; Figure 3B). A morphotype analysis of *A. fumigatus* in unfused phagolysomes found that the percentage containing spores reduces from 74.57% at 5 h to 69.86% at 20 h, but then rises up to 77.86% at 24 h, while spore germination within unfused phagosomes underwent the opposite trend (*p* < 0.0001; Figure 3C). Spore digestion and germling escape from alveolar epithelial cells over the infection time course may account for these trends. 

**Figure 4:**
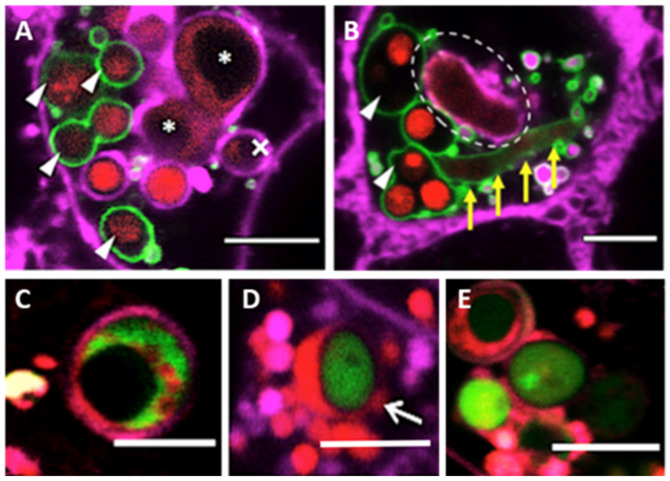
Killing of *A. fumigatus* within A549 alveolar epithelial cell lines. (**A**) Single airway epithelial cell containing digested (triangles) and killed germlings (asterisks) within phagolysosomes. The cross symbol refers to the crescent feature after digestion of a spore within a phagosome. (**B**) Hyphal killing occurs within phagolysosomes (yellow arrows) and within phagosomes (indicated by dashed circle). (**C**) Killing of *A. fumigatus* within the acidified phagosome. (**D**) Residence of *A. fumigatus* spore co-localized within acidic niche for more than 18 h. (**E**) Spore swelling within the acidified phagosome 24 h of inoculation. *Color code for panels A and B:* Magenta = epithelial cells membrane (CellMask Deep Red), Green = lysosomes (GFP-LAMP1), red = *A. fumigatus* (MFIGRag29). *Color code for panels C–E*: Magenta = epithelial cells membrane (CellMask Deep Red), Red = Acidic organelles (RFP-Lysotracker), Green = *A. fumigatus* (MFIGGFP4, Table S1). Scale Bar = 5 μm.

Additionally, our fluorescence image data suggests that phagolysosomes can digest most of the internalized spores and germlings at 20 h post-infection, as shown by the loss of MFIGRag29 fluorescence in fungal cells (Figure 4). However, even when phagosome-lysosome fusion failed, spores could still be killed during acidification of the phagosome (Figure 4). Overall, our results indicate that efficiency of fungal phagocytosis by A549 alveolar epithelial cells, to prevent spore germination and further epithelia invasion, mainly depends on the level of lysosomes that fuse with spore-containing phagosomes. 

### 3.2. Bronchial Epithelial Cell Phagolysosomes Demonstrate Increased Fungal-Killing, Compared to Alveolar Cells

Live-cell confocal microscopy was used to quantitatively compare phagolysosome dynamics, during *A. fumigatus* spore internalization, within bronchial (16HBE) and alveolar (A549) epithelial cell lines. These cell lines represent the epithelial lining of the upper and lower airways, both critical in the pathophysiology of aspergillosis [49,50].

**Figure 5:**
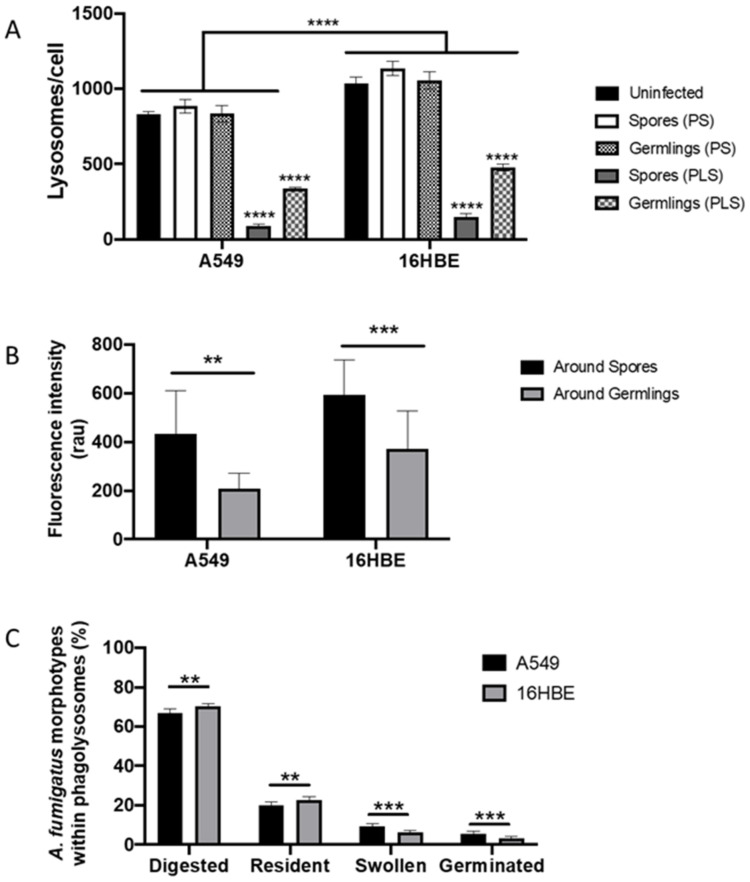
Phagolysosome formation is critical to prevent *A. fumigatus* growth within airway epithelial cells. (**A**) Changes in the number of lysosomes per cell in 16HBE and A549 lung epithelial cells challenged and unchallenged with *A. fumigatus* spores. Asterisks represent differences between spores or germlings compared with uninfected for each cell line. Lines represent differences between groups for each cell line. (**B**) Intensity of relative LAMP1-GFP fluorescence intensity around spore or germlings-containing phagolysosomes of A549 and 16HBE lung epithelial cells. Asterisks represent differences between spores and gemlings for each cell line. (**C**) Fate of *A. fumigatus* spore within phagosomes (PS) and phagolysosomes (PLS) of A549 and 16HBE lung epithelial cells. Asterisks represent differences for each morphotype between cell lines. Data represents mean and standard deviation of three biological replicates assayed in technical triplicates. (** *p* < 0.01; *** *p* < 0.001; **** *p* < 0.0001).

The number of unfused lysosomes around spores and germlings within a phagosome was significantly higher than unfused lysosomes around spores or germlings localized within phagolysosomes in both, A549 alveolar (150 ± 18.91 vs. 89.80 ± 13.63 lysosomes/A549 cell; *p* < 0.0001) and 16HBE bronchial epithelial cells (479.60 ± 22.99 vs. 336.60 ± 10.24 lysosomes/16HBE cell, *p* < 0.0001). There were no statistically significant differences between the number of lysosomes in uninfected cells and cells containing a spore or germling within a phagosome, prior to the initiation of lysosome fusion, suggesting that fungal uptake does not induce a change in lysosome production in both epithelial cell types (Supplementary Figure S1 and Figure 5A). However, the number of lysosomes per cell were significantly higher in 16HBE cells than in A549 cells (*p* < 0.0001) (Figure 5A).

To characterize phagosome-lysosome fusion rate during *A. fumigatus* infection, the cumulative intensity of fluorescence emitted by lysosomal GFP-LAMP1 labelled proteins entering the phagosomal membrane was measured from 4 to 16 h post-infection of A549 and 16HBE lung epithelial cells [35]. For each fungal morphotype (spore or germling), the mean fluorescence intensity for completely fused lysosomes was measured at the phagolysosome. The mean intensity of GFP-LAMP1 fluorescence around spore-containing phagolysosomes was significantly higher than germling-containing phagolysosomes in both lung epithelial cell lines (Figure 5B).

To test whether differences in the efficiency of phagosome lysosome fusion between both lung epithelial cell lines prevented spore germination or conversely, spore germination prevents phagolysosome maturation; the germination time of *A. fumigatus* spores in phagosomes and phagolysosomes, were compared in 16HBE and A549 cells. While there were no differences in the germination time within unfused phagosomes between epithelial cell lines; germination was significantly delayed within phagolysosomes of 16HBE bronchial epithelial cells compared to A549 alveolar epithelial cells (*p* < 0.05, Figure S2).

To further define if increased phagosome-lysosome fusion rate by the bronchial epithelium correlates with inhibition of *A. fumigatus* morphogenesis within phagolysosomes, the development and fate of internalized *A. fumigatus* spores within A549 and 16HBE phagolysosomes were followed in a 16 h time course (Figure 5C). Although most internalized spores were digested at 16 h post-infection (A549 = 66.66% and 16HBE = 70.17%; *p* < 0.05), a significant proportion of internalized *A. fumigatus* spores remained within phagolysosomes for more than 16 h within A549 (20.04%) and 16HBE (22.59%) cells. Overall, 9.38% of internalized spores in A549 cells and 6.17% of internalized spores within 16HBE cells were able to swell, while 3.52% (16HBE) and 5.62% (A549) germinated within the phagolysosome (*p* < 0.0001). Similarly, the proportion of killed spores in both cell lines were higher than living spores, where the proportion of killed spores within 16HBE cells (67.19%) was significantly higher than A549 cells (57.61%) (Figure S3). Altogether, these data suggest that the improved antifungal efficiency of the bronchial epithelium relies on an increased fusion rates of spores contained in phagosomes.

### 3.3. Phagolysosome Maturation Relies on A. fumigatus Cell Wall Integrity

We hypothesized that differential killing of spores in bronchial and alveolar cells results from differences in the interaction of host with pathogen factors such as hydrophobins and melanins. Therefore, the efficacy of epithelial cells to kill *A. fumigatus rodA, rodB* and *pksP* null mutants was analyzed at 6 h post infection. Comparative analyses demonstrated that *A. fumigatus pksP* null mutant was the most susceptible to killing (8–10% survival decrease) compared to the parental *A. fumigatus* strain within the phagolysosomes of both A549 and 16HBE cells (*p* < 0.001). There were no differences in the potential of the lung epithelium to kill the rodetless mutants compared to the parental strain. However, *A. fumigatus* mutants deficient in all tested cell wall components were more susceptible to killing within 16HBE cells (Figure S4; *p* < 0.01), and this may correlate with increased phagosome-lysosome fusion or acidification in this cell line.

**Figure 6:**
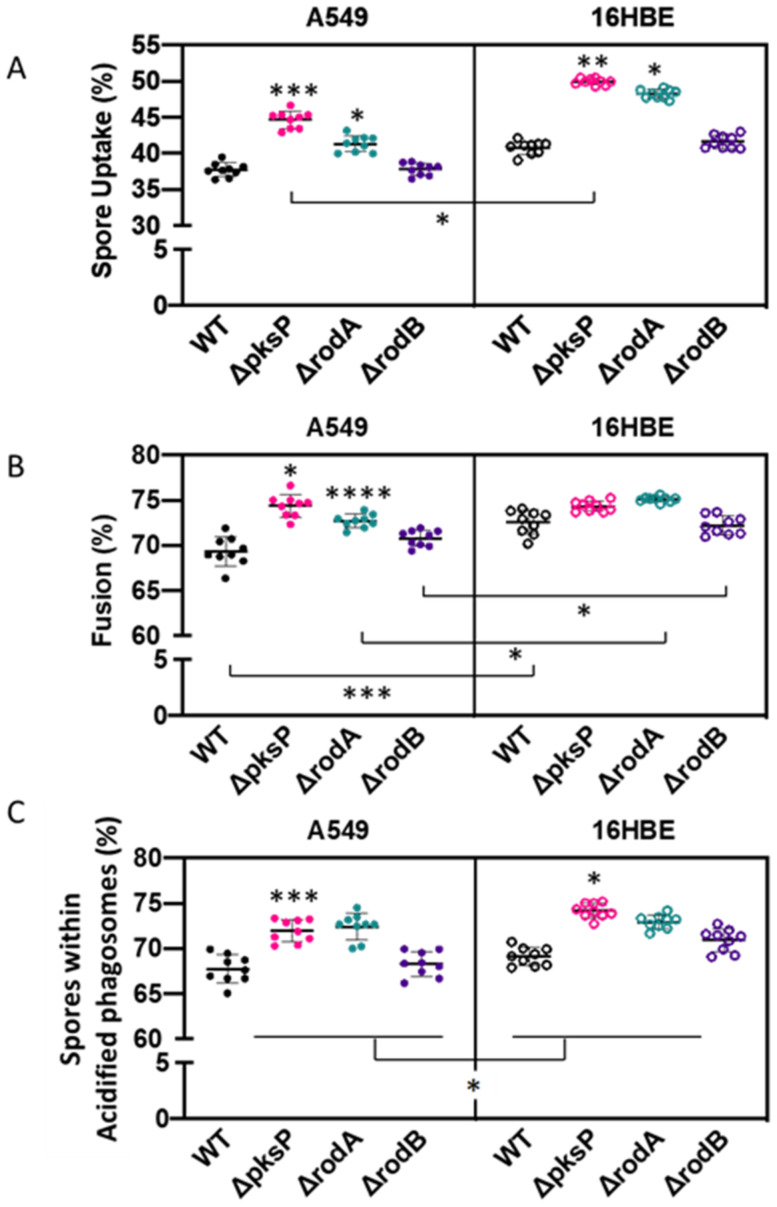
Impact of *A. fumigatus* cell wall mutants in (**A**) spore uptake, (**B**) phagolysosome fusion and (**C**) phagosome acidification of A549 and 16HBE airway epithelial cells at 3 h post-infection. Asterisks represent differences vs. wild type for each mutant within cell lines. Lines represent differences between cell lines for each mutant. Data shown as mean ± standard deviation of three biological replicates assayed in technical triplicates (* *p* < 0.05, ** *p* < 0.01, *** *p* < 0.001, **** *p* < 0.0001).

To validate our observations and the throughput potential of our imaging approach, we comparatively determined differences in spore uptake, phagosome-lysosome fusion and acidification between 16HBE and A549 lung epithelial cell lines when challenged with *A. fumigatus* cell wall mutants at 3 h post-infection. This time point was chosen as our initial experiments suggested complete phagosome-lysosome fusion at 6 h post-infection of A549 cells (Figure 1). Spore uptake was significantly increased in mutant *A. fumigatus* (Δ*pksP* and Δ*rodA*) compared to wild type by both 16HBE and A549 lung epithelial cells (Figure 6A; *p* < 0.05). There were no significant differences in the percentage of spore uptake between the parental strain and Δ*rodB* strains by both cell lines. Although no significant differences were found between wild type and Δ*rodB* spore uptake by A549 and 16HBE lung epithelial cells (*p* = 0.16), uptake of the Δ*rodA* and Δ*pksP* mutants were significantly higher in 16HBE compared to A549 lung epithelial cells (*p* < 0.05).

There were no significant differences in the percentage of internalized *A. fumigatus* spores within phagolysosomes of 16HBE cells for any of the cell wall mutants tested. However, phagolysosome development was most efficient when containing *A. fumigatus pksP* and *rodA* null mutants compared to parental spores in A549 cells (*p* < 0.05). Overall, the percentage of phagolysosomes containing wild type, Δ*rodA* and Δ*rodB* spores was significantly higher (*p* < 0.05) in 16HBE (69.34%, 72.70% and 70.80%, respectively) cells compared to A549 (72.55%, 75.11% and 72.22%, respectively; Figure 6B).

It was previously observed that phagosome acidification may act as a rescue mechanism capable of killing *A. fumigatus* spores when lysosome fusion is complete, or it fails to occur [35]. Only a small increase in the percentage of *A. fumigatus* Δ*pksP* spores were colocalized within acidified compartments, compared to wild type, for both 16HBE and A549 cell lines (*p* < 0.05) at 3 h post-infection (Figure 6C). Overall, phagosomes acidified more rapidly in 16HBE than A549 cells (*p* < 0.05). These data indicate that a high content live-cell confocal microscopy is able to distinguish even subtle variations in phagosome maturation caused by *A. fumigatus* cell wall integrity factors.

Overall, the percentage of agreement to call spores within phagolysosomes or acidified compartments was 87.5% and 89.5% respectively, when using manual or automated methods (Supplementary Tables S2 and S3).

### 3.4. Validation of High Content Live-Cell Imaging on vATPase Silenced Lung Epithelial Cells

**Figure 7:**
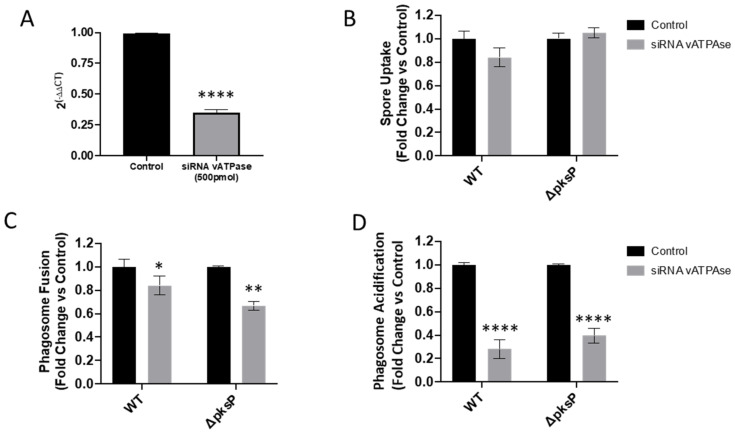
Impact of vATPase siRNA silencing of A549 alveolar epithelial cells in (**A**) vATPAse gene expression, (**B**) *A. fumigatus* spore uptake, (**C**) phagolysosome fusion and (**D**) phagosome acidification by A549 and 16HBE cells at 3 h post infection. Asterisks represent differences vs. control. Data shown as mean ± standard deviation of three biological and technical triplicates (* *p* < 0.05, ** *p* <0.01, **** *p* <0.0001).

In order to investigate the role of vATPase (ATP6V0A2 gene) upon fusion and acidification of *A. fumigatus* wild type and Δ*pksP* spore-containing phagosomes. Expression of vATPase was decreased by 66% using siRNA (Figure 7A) in A549 epithelial cells. This reduction of vATPase expression did not modify *A. fumigatus* uptake compared to untreated cells (Figure 7B). Lysosome fusion to spore-containing phagosomes was significantly reduced in ATP6V0A2-siRNA treated A549 cells compared to untreated cells (*p* < 0.05, Figure 7C). Additionally, reduced vATPase expression disguised the impact of this *A. fumigatus* mutant in fusion, as described by others (Figure 7B). Similarly, as expected, the reduction of ATP6V0A2 expression strongly limited the capability of spore-containing phagosomes to be acidified compared to control non-siRNA treated A549 epithelial cells (Figure 7D). These results demonstrate that our high content confocal imaging approach can detect host and pathogen factors contributing to *A. fumigatus* survival within airway epithelial cells.

## 4. Discussion

The airway epithelium is the first point of contact of *A. fumigatus* spores with the host. In-vitro confrontation assays are typically visualized by microscopy, and manually observed and quantified, which is time consuming, where user variability and error occur across biological replicates. Additionally, these methodologies typically use macrophages as the model cell type to study fungal-host interaction and are performed using fixed samples which can alter the outcome of the interaction of *Aspergillus* with the host [51,52,53,54,55]. Here, we have combined a live-cell labelling strategy with automated 4D live-cell imaging to visualize and measure critical events required for *Aspergillus* clearance by the airway epithelium. We have used this methodology to determine differences in *A. fumigatus* fate within two of the most commonly used airway epithelial cell lines, A549 (alveolar) and 16HBE (bronchial), and to explore the role of fungal cell wall components on these processes.

Lysosomes are cytoplasmic organelles which play an essential function in the recognition and killing of intracellular infective microorganisms [56]. Recently a significant number of live-cell imaging technologies have been developed to quantify lysosome moieties using fluorescence probes (for review [57]), but little is known about lysosome dynamics during active fungal infection. Using GFP-LAMP-1 epithelial cell transduction, we determined for the first time the rate of lysosome depletion upon *A. fumigatus* spore infection. Our results indicate that once a spore is localized within the phagosome, of either A549 or 16HBE airway epithelial cells, lysosome fusion is completed within 60 min. Additionally, cytoplasmic lysosomes were depleted to those phagosomes containing *A. fumigatus* germlings, suggesting the need for a higher number of lysosomes to be fused to the phagosome in order to limit germling growth and prevent non-lytic escape [35]. Moreover, lysosome positioning contributes to phagosome maturation and antigen presentation in dendritic cells challenged with bacterial lipopolysaccharide. It is therefore likely that an increased exposure of antigenic moieties in *A. fumigatus* germlings facilitates lysosome recruitment [58,59].

To further characterize the dynamics of phagolysosome maturation during *A. fumigatus* morphogenesis within airway epithelial cells, GFP-LAMP-1 accumulation was indirectly quantified as fluorescence intensity around spores or germlings. A reduction of GFP-LAMP1 accumulation around phagolysosomes containing germlings was observed for both alveolar and epithelial cells, suggesting that some fungal factors secreted during *A. fumigatus* growth, such as proteases, may degrade LAMP1, facilitating fungal survival as previously described for other pathogens [60]. Nevertheless, this suboptimal lysosomal activity toward phagolysosome germlings was sufficient to better contain fungal germination, compared to unfused phagosomes. Overall, >10% of the internalized spores were able to swell or germinate within phagolysosomes, suggesting morphogenesis as the mechanisms of host escape, as previously described in *Candida albicans* and *Legionella pneumophila* [61,62].

The rate of phagosome-lysosome fusion determines the microbicidal potential of these organelles while maintaining cell homeostasis [34]. Respiratory pathogens have developed different strategies to prevent phagosome quelling by either modifying uptake, phagosome-lysosome fusion rate or acidification. *A. fumigatus* cell wall melanins and hydrophobins protect the spores from the host defence recognition. The roles of *A. fumigatus pksP, rodA* and *rodB* in phagolysosome maturation and intracellular killing by both macrophages and epithelial cells have previously been described using LAMP-1 antibody labelled host cells [19,27,44]. In order to validate the utility of our live-cell automated quantitative approach, the percentage of internalized spores fused with phagosomes was determined at 3 h post-infection. In accordance with previous studies, phagosome-lysosome fusion was found to increase in *A. fumigatus pksP* and *rodA* null mutants in both A549 and 16HBE airway epithelial cells, which is linked with a significant decrease in fungal survival [5,39,44,63,64,65,66]. In A549 cells, no difference in spore uptake or phagosome-lysosome fusion was observed in the *rodB* null mutant compared to wild type, even though both processes were enhanced in bronchial epithelial cells [55]. This high-resolution live-cell imaging approach has also enabled us to observe digested *A. fumigatus* spores within airway epithelial cell phagolysosomes for the first time, as reported for spores contained within macrophages [14].

Alongside phagolysosome formation, the successful destruction of an engulfed pathogen and the presentation of antigens to activate immunological responses requires an appropriate level of acidification of the phagosomal lumen [67]. Phagosome acidification in *A. fumigatus* in-vitro infection models has previously been determined using either acridine orange or lysotracker. We and others have described that *A. fumigatus* spore localization within acidified phagosomes occurs early after infection [27,35,66]. Here, we were able to additionally follow that most of the spores contained within fully acidified phagosomes from airway epithelial cells were killed. It is likely that spore killing within acidified phagosomes is due to the permeation of acids and enzymes through the spore wall, with the formation of pores on the spore surface as reported for other model systems [55]. To determine the suitability of our image rendering approach to quantify phagosome acidification, the capability of *A. fumigatus* cell surface mutants to acidify phagosomes was determined and compared by manual counts. As previously described, functionality of *pksP* and *rodA* is critical to prevent phagosome acidification in a process regulated by vATPase functionality [68,69]. Altogether, our results demonstrate the suitability of this imaging analyses pipeline to quantify critical aspects of *A. fumigatus* endocytic trafficking within epithelial cells.

Direct comparisons of *Aspergillus* fate in A549 and 16HBE cell lines have not been previously addressed (for review [70]), despite differential activation with lipopolysaccharide [46], *Fusobacterium* [71], glucocorticoids [72] and cigarette smoke [73] exposure. Overall, 16HBE cells were able to take up the *A. fumigatus* strains Δ*pksp* and Δ*rodA* more efficiently than A549, which may be linked with increased expression of mucus on the epithelia [74]. Additionally, improved phagosome maturation in 16HBE cells compared to A549 correlated with a 10% increment in fungal killing. Despite the inhibition of phagosome-lysosome fusion by the expression of *pksP* or *rodA* in *A. fumigatus* in A549 cells, this was not observed in 16HBEs cells, as reported for amoeba and macrophages [55]. Differences across fungal cell mutants in their interaction with 16HBE cells may be disguised due to their increased uptake, phagosome-lysosome fusion and acidification compared to A549 cells, suggesting that further time points are required to find functional differences, if there are any. However the evidence for enhanced antifungal effectiveness of the bronchial epithelium, compared to alveolar epithelial cells, is building [75]. We also confirmed the vATPase requirement for optimal phagosome acidification of both phagosome containing melanised and non-melanised fungi, as reported in macrophages [68].

In conclusion, we have interrogated the use of a high-resolution live-cell confocal quantitative imaging approach to characterise crucial events occurring during the interaction of internalised *A. fumigatus* spores and lung epithelial cells. These automated quantification methods, which have been used in other experimental systems [35,37,48,68,76,77], supports robust statistical analyses to determine the efficiency of *A. fumigatus* morphogenesis within phagosomes and phagolysosomes in epithelial cells, in a similar way to using manual counts [78], and allows to determine fungal factors that modulate this process. Additionally, our results demonstrate that *A. fumigatus* fate within the airway epithelium depends on the cell type, but is likely also conditioned by other factors such as multiplicity of infection. It has been widely described that uptake of *A. fumigatus* by airway epithelial cells increase on a time-dose dependent manner, and it is therefore likely that the time points described in this work are not optimum when using a smaller inoculum [19,21,27,28,32,70]. The methodology described here could be used to study the interaction of other fluorescent pathogens within primary lung epithelia cells or other host cells.

## Data Availability

The data presented in this study are available on request from the corresponding authors.

## Supplementary Materials

The following are available online at https://www.mdpi.com/article/10.3390/jof7060454/s1, Figure S1: Variation in the number of lysosomes during phagolysosome development Figure S2: Germination of *A. fumigatus* spores within phagolysosomes is delayed compared to germination time within phagolysosomes. Figure S3: Proportion of killed spores within acidic phagosomes of 16HBE and A549 epithelial cell. Figure S4: Percentage of *A. fumigatus* spore killed within epithelial cells. Table S1: *A. fumigatus* strains used in this study. Table S2: Comparison of automated and manual spore calling results within phagolysosomes (PL) in five independent images. Cohen’s K respresents Kappa test. Table S3: Comparison of automated and manual spore calling results within acidified phagosomes (AF) in five independent images. Cohen’s K respresents Kappa test.
