# Supplementary material for: Characterisation of Aspergillus fumigatus Endocytic Trafficking within Airway Epithelial Cells Using High-Resolution Automated Quantitative Confocal Microscopy
Source: J Fungi (Basel). 2021 Jun 7;7(6):454. doi: 10.3390/jof7060454 (PMC8229978; doi:10.3390/jof7060454)
Supplement: Supplementary file 1 [file jof-07-00454-s001.zip › jof-1243996-supplementary.pdf]

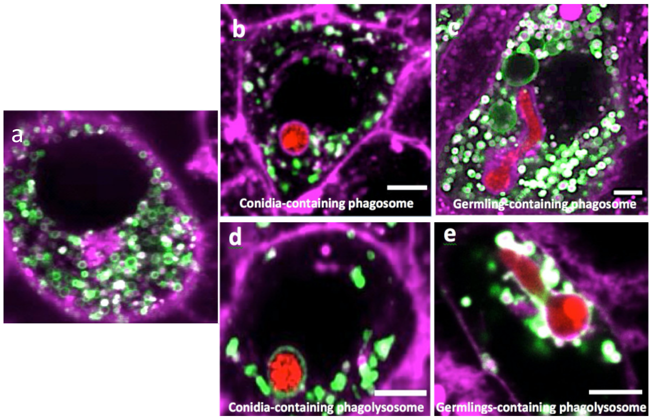

Figure S1. Variation in the number of lysosomes during phagolysosome development. Number and locations of lysosomes in (a) Uninfected, (b) Phagosome-containing conidia, (c) Germling-containing phagosome, (d) Conidia-containing phagolysosome and (e) Germling-containing phagolysosome of A549 alveolar epithelial cells. Red = conidia, green = lysosome, magenta = plasma membrane. Scale Bar = 5 μm. Magenta = Cell Mask Deep Red, Green = GFP-LAMP1, Red = MFIGRag29 (Table S1).

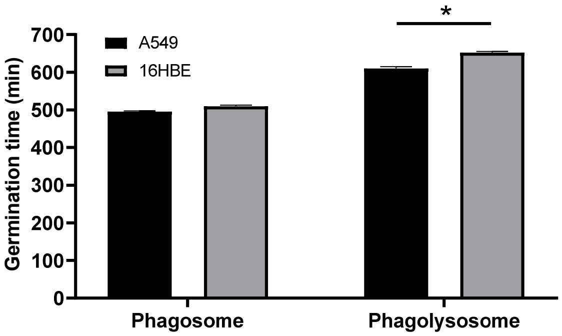

Figure S1. Germination of *A. fumigatus* spores within phagolysosomes is delayed compared to germination time within phagosomes. Data shown as mean ± standard deviation of three biological and technical replicates. . * $P < 0.05$. Asterisks represent differences between cell lines.

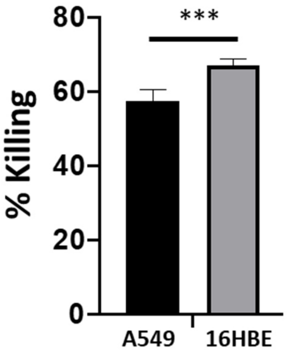

Figure S3: Proportion of killed spores within acidic phagosomes of 16HBE and A549 epithelial cell. Data shown as mean ± standard deviation of three biological and technical replicates. Asterisks represent differences between cell lines (*** $P < 0.001$).

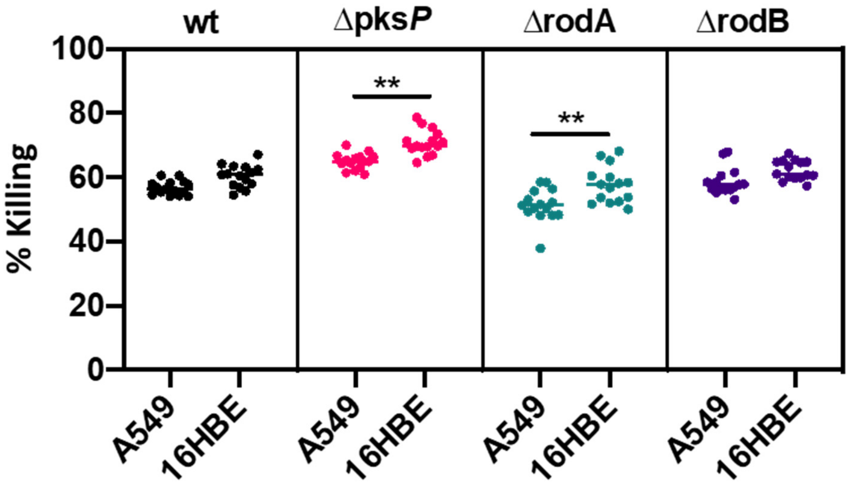

Figure S4: Percentage of *A. fumigatus spore* killed within epithelial cells. Data represents Mean and standard deviation of three biological and technical replicates. Asterisks represents differences between cell lines. (***P* < 0.001).

Table S1. *A. fumigatus* strains used in this study.

Strain	*Function*	*Gene name*	*Parental strain*
A1160	-	-	*CEA10 (CBS1144.89)*
MFIGGFP4	*Cytoplasmic GFP*	-	*A1163 (FGSC A1163) (pyrG⁻::βtub-GFP,pyrG⁺)*
MFIGRag29	*Cytoplasmic RFP*	-	*A1163 (FGSC A1163) (pyrG⁻::βtub-TurboRFP635,pyrG⁺)*
A1160_ΔrodA	*Rodlet layer protein A*	*AFUB_057130*	*A1160, ΔKu80 pyrG⁺*
A1160_ΔrodB	*Rodlet layer protein B*	*AFUB_016640*	*A1160, ΔKu80 pyrG⁺*
A1160_ΔpKsP	*Polyketide synthase required for DHN-melanin*	*AFUB_033290*	*A1160, ΔKu80 pyrG⁺*

Table S2. Comparison of automated and manual spore calling results within phagolysosomes (PL) in five independent images. Cohen's K rrespresents Kappa test.

	**Manual**			**Automated**			**Cohen's K**	**% Agreement**
	**Total**	**Within fused PL**	**% Fusion**	**Total**	**Within fused PL**	**% Fusion**		
**1**	25	12	48	28	15	53	0.84	92.68
**2**	*27*	*6*	*22*	*23*	*10*	*43*	0.65	85.18
**3**	44	17	38	42	19	45	0.82	91.12
**4**	20	4	20	35	10	28	0.42	76.92
**5**	39	16	41	31	14	45	0.74	91.30
**Total**	**155**	**55**		**159**	**68**		**0.7**	**87.5%**
**% Fusion**		**35%**			**42%**			

Table S3. Comparison of automated and manual spore calling results within acidified phagosomes (AF) in five independent images. Cohen's K respresents Kappa test.

	**Manual**			**Automated**			**Cohen's K**	**% Agreement**
	**Total**	**Within AP**	**%**	**Total**	**Within AP**	**%**		
**1**	22	5	23	27	7	26	0.79	93.10
**2**	27	8	30	23	5	21	0.57	84.73
**3**	35	10	28	37	6	16	0.64	89.13
**4**	23	2	8	19	2	10	0.43	84
**5**	38	6	16	34	7	20	0.90	97.56
**Total**	**145**	**31**		**140**	**27**		**0.66**	**89.7**
**% Acidified**		**21%**			**19%**			
